# How lifespan associated genes modulate aging changes: lessons from analysis of longitudinal data

**DOI:** 10.3389/fgene.2013.00003

**Published:** 2013-01-22

**Authors:** Anatoliy I. Yashin, Konstantin G. Arbeev, Deqing Wu, Liubov S. Arbeeva, Alexander Kulminski, Igor Akushevich, Irina Culminskaya, Eric Stallard, Svetlana V. Ukraintseva

**Affiliations:** Center for Population Health and Aging, Duke UniversityDurham, NC, USA

**Keywords:** age trajectories, physiological variables, longevity genes, genetic dose, integrative genetic mortality model

## Abstract

**Background and Objective:** The influence of genes on human lifespan is mediated by biological processes that characterize body's functioning. The age trajectories of these processes contain important information about mechanisms linking aging, health, and lifespan. The objective of this paper is to investigate regularities of aging changes in different groups of individuals, including individuals with different genetic background, as well as their connections with health and lifespan. **Data and Method:** To reach this objective we used longitudinal data on four physiological variables, information about health and lifespan collected in the Framingham Heart Study (FHS), data on longevity alleles detected in earlier study, as well as methods of statistical modeling. **Results:** We found that phenotypes of exceptional longevity and health are linked to distinct types of changes in physiological indices during aging. We also found that components of aging changes differ in groups of individuals with different genetic background. **Conclusions:** These results suggest that factors responsible for exceptional longevity and health are not necessary the same, and that postponing aging changes is associated with extreme longevity. The genetic factors which increase lifespan are associated with physiological changes typical of healthy and long-living individuals, smaller mortality risks from cancer and CVD and better estimates of adaptive capacity in statistical modeling. This indicates that extreme longevity and health related traits are likely to be less heterogeneous phenotypes than lifespan, and studying these phenotypes separately from lifespan may provide additional information about mechanisms of human aging and its relation to chronic diseases and lifespan.

## Introduction

The influence of genes on lifespan is mediated by biological variables which integrate responses to numerous external and internal challenges to maintain functioning of an organism's life supporting and reproductive machineries. Some of these variables are measured in longitudinal studies of aging, health and longevity. To mediate genetic effects on lifespan, these variables have to be associated with lifespan as well. Such associations have been established in epidemiological studies for a number of physiological variables where their roles as risk factors for all-cause mortality or chronic degenerative diseases have been investigated. Among many other variables, body mass index (BMI), diastolic blood pressure (DBP), serum cholesterol (SCH), and ventricular rate deserve particular attention because their typical average age trajectories are non-monotonic and their associations with all-cause mortality have been widely studied. Specifically, the effect of BMI on risk of diseases and mortality was intensively studied in connection with metabolic syndrome. Freedman et al. (2006) showed that the connection between BMI and mortality risks is generally J-shaped for both genders and different age groups. The authors also found that this risk function changes with age. The relationships between mortality risk and BMI were also assessed (see Zhou, 2002; Gu et al., 2006; Gelber et al., 2007; Klenk et al., 2009, among others).

The connection between DBP and all-cause mortality risk has been investigated to better understand factors and mechanisms of cardiovascular diseases (CVD) (Cruickshank, 1988, 2003; Staessen, 1996). Special attention has been paid to the J-shape of the risk function (see Isles and Hole, 1992; Alderman, 1996; Cruickshank, 2003; Messerli and Panjrath, 2009; Grassi et al., 2010, among others). Franklin et al. (2001) studied changes in this risk function with age. Boshuizen et al. (1998) studied the connection of blood pressure and mortality risk among the elderly. Questions of optimal blood pressure were discussed by Onrot (1993) and Townsend (2005), among others.

Anderson et al. (1987) evaluated the connection between SCH and mortality using 30 years of follow-up data from the Framingham Heart Study (FHS). The authors found that a 1% increase in total cholesterol produced a 2% increase in coronary heart disease incidence among individuals between 60 and 70 years of age. Kronmal et al. (1993) found that the relationship between total cholesterol level and all-cause mortality was positive at age 40 years, negligible at ages 50–70 years, and negative at age 80 years. Manolio et al. (1992) and Weverling-Rijnsburger et al. (1997, 2003) showed that CVD in old age was independent of total SCH levels. Weverling-Rijnsburger et al. (1997) proposed that this could be a result of selective mortality of those with the highest cholesterol levels in middle age. Weverling-Rijnsburger et al. (1997) and Schatz et al. (2001) showed that low total SCH levels are associated with higher all-cause mortality in the oldest old. The relationship between SCH and all-cause mortality was also studied by Chyou and Eaker (2000) and Li et al. (2004a,b), among others.

The effects of resting heart rate (also called ventricular rate, VR) on cardiovascular mortality have been discussed in Kannel et al. (1987). Mensink and Hoffmeister (1997) and Benetos et al. (1999) investigated the effects of resting heart rate on all-cause mortality. The connection between heart rate and mortality in the elderly has also been investigated by Cacciatore et al. (2007). Kuzuya et al. (2008) found a J-shaped relationship between resting pulse rate and all-cause mortality in community dwelling older people with disabilities. Böhm et al. (2012) showed that resting heart rate in clinical conditions is associated with all-cause mortality, disability, and cognitive decline.

The results described above indicate that studying aging related changes in physiological variables as well as genetic factors involved in their regulation using available longitudinal data could make substantial contributions to clarifying mechanisms linking aging, health, and longevity in humans, and provide useful insights into alternative strategies for improvement of people's health by postponing the aging process (Kristjuhan, 2012). Note that none of the studies mentioned above performed either systematic analyses of age patterns of corresponding variables, or their roles in mediating genetic influences on lifespan. The longitudinal data on aging, health, and lifespan collected in the FHS contain valuable information on biennial measurements of these physiological variables during the life courses of study participants, detailed data on their genetic background, as well as data on health and survival outcomes which can be used for testing the ability of these variables to mediate genetic influences on lifespan.

In this paper we evaluate and discuss the properties of the average age trajectories of the four physiological indices described above, evaluate the connections of the shapes of these trajectories to lifespan, and individuals' health status. We also evaluate how the different doses of pro-survival alleles carried by study participants are associated with the age patterns of their physiological variables. Using a stochastic process model (Yashin et al., 2007a), we evaluate hidden components of the process that drive aging related changes in physiological variables.

## Data and methods

### The framingham heart study (FHS) data

The FHS Original cohort was launched at Exam 1 in 1948 and has continued with biennial examinations to the present (30 exams to date; data from exams 1–28 were available for this study). The FHS Original cohort consists of 5209 respondents (55% females) aged 28–62 years at baseline residing in Framingham, Massachusetts, between 1948 and 1951. Nearly all subjects were Caucasians. The examination included an interview, physical examination, and laboratory tests. Individual information on the SNP genotyping and phenotypic traits collected in the Framingham Study was obtained through the dbGaP website. The data on phenotypic traits collected in the Original FHS cohort over 60 years and relevant to our analyses include: ages at disease onsets for cancer, CVD and diabetes, causes of death, lifespan, and various factors that may affect disease risk and prognosis including BMI (data available at exams 1, 4, 5, and 10–28), DBP (exams 1–28), SCH (exams 1–11, 13–15, 20, and 22–28), VR (exams 4–28), age at exam, sex, birth cohort, and smoking status (exams 1, 4, 5, 7–15, and 17–28).

The occurrence of diseases (CVD and cancer) and death (including information on the cause of death coded as death from cancer, CVD, and all other or unknown causes) has been followed through continuous surveillance of hospital admissions, death registries, clinical exams, and other sources, so that all the respective events are included in the study. We used data on first occurrence of CVD (defined by the FHS investigators as having any of the following: coronary heart disease, intermittent claudication, congestive heart failure, or stroke/transient ischemic attack) and cancer from the follow-up data, and data on current diabetes status (defined by the FHS investigators as a level of blood glucose exceeding 140 mg/dl and/or taking insulin or oral hypoglycemics) in exams in analyses involving the onset of “unhealthy life” (see below).

**Figure 1:**
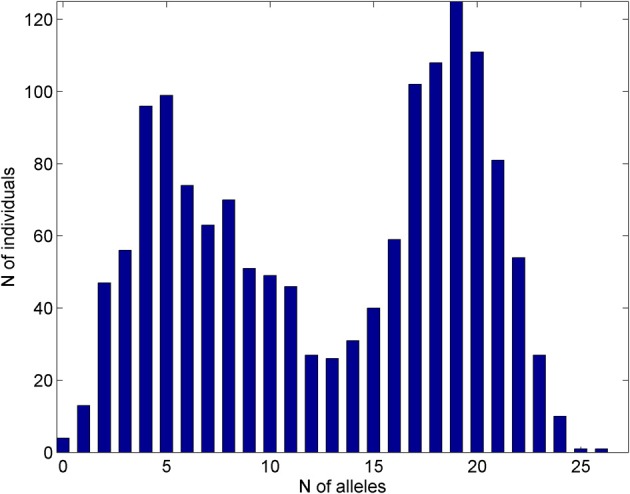
**Distribution of the numbers of pro-survival alleles out of 27 of such alleles selected in Yashin et al. (2012c) in the sample of genotyped individuals in the FHS Original cohort**.

We also used information about the distribution of 27 “pro-survival” alleles among participants of the FHS Original cohort. Figure 1 shows distribution of the numbers of these alleles in the sample of genotyped individuals in the FHS Original cohort. These genetic variants showed highly significant joint influence on lifespan in our recent study (Yashin et al., 2012c). They were selected out of 550,000 SNPs in 1471 genotyped participants of the FHS Original cohort as described in Yashin et al. (2012c). Table 1 below shows essential information about the 27 SNPs and their closest genes. More detailed information about the genes closest to the 27 SNPs, including their biological functions and links to aging and disease phenotypes, as found in current literature, is provided in Yashin et al. (2012c). In brief, of the 27 SNPs, 16 were located within functioning genes, and all these SNPs but one were intronic. Overall, these genes have been linked in the literature to multiple phenotypes, although they were more often involved in cancer and brain disorders. While some of the genes, in which the selected SNPs are located, may produce specific physiological effects, none of the 27 SNPs identified in Yashin et al. (2012c) has been found so far to be individually significantly associated with BMI, DBP, SCH, or VR.

**Table 1:** **Essential characteristics of the 27 pro-survival SNPs from Yashin et al. (2012c)**.

**SNP**	**MAF %**	**Chr**	**Relation to gene**	**Distance to gene**	**Closest gene**	**Gene/protein description**	**Major biological processes or health disorders, in which respective genes were shown/suggested to be involved**
rs4648884	27.7	1	INTRONIC	0	RUNX3: runt-related transcription factor 3	Can either activate or suppress transcription; interacts with other transcription factors	Apoptosis; tumor suppression; immune response; asthma
rs3120819	42.1	1	INTERGENIC	−84906	RP11-149P14.1	Pseudogene	
rs1974676	35.4	2	INTRONIC	0	HPCAL1: hippocalcin-like 1	Member of neuron-specific calcium-binding proteins family	Brain information processing; CVD, asthma (?)
rs432203	38.0	2	INTRONIC	0	TGF alpha: transforming growth factor, alpha	A mitogenic polypeptide able to bind to the EGF receptor and act synergistically with TGF beta to promote cell proliferation	Cell proliferation; brain response to damage; cancer; stroke (?)
rs12623542	36.7	2	NON CODING	0	AC131097.3		
rs13008689	23.2	2	INTERGENIC	−153466	AC011747.3		
rs1834497	44.7	3	INTRONIC	0	CLSTN2: calsyntenin 2	Postsynaptic membrane proteins with highest levels in GABAergic neurons; expressed in the medial temporal lobe	Brain information processing; memory formation; AD
rs9876781	34.9	3	NON CODING	0	RP11-24C3.2	Closest gene: ATR interacting protein (ATRIP)	Cell cycle arrest/ replicative senescence
rs10937739	41.9	4	INTRONIC	0	PPP2R2C: protein phosphatase 2, regulatory subunit B, gamma	Serine/threonine phosphatase 2; is abundant in brain; can modify MAPK activity;	Apoptosis; memory; brain disorders; cancer, CVDs (?)
rs1205035	36.2	6	INTERGENIC	−109050	RP1-223B1.1		
rs3800358	25.9	6	3PRIME_UTR	0	BTBD9: BTB domain containing 9	Involved in protein-protein interactions	Sleep disorders
rs10256972	36.5	7	INTRONIC	0	C7orf50: chr7 open reading frame 50		
rs1327533	25.3	9	INTRONIC	0	SVEP1: EGF and pentraxin domain containing 1	A multi-domain protein: Sushi, CCP, vWF-A, EGF/EGF-like calcium binding, and pentraxin domains	Cell adhesion; muscle growth and regeneration
rs2590504	37.3	9	DOWN-STREAM	−6153	KIAA0649	Is a 1A6/DRIM ("Down-regulated in Metastasis") interacting protein with oncogenic characteristics	Cell proliferation; cancer
rs10819510	26.5	9	INTERGENIC	−37267	RP11-65J3.3		
rs739401	32.4	11	INTRONIC	0	CARS: cysteinyl-tRNA synthetase	Catalyzes tRNA aminoacylation; located near imprinted gene domain in a tumor-suppressor gene region	Cancer; diabetes
rs2370413	35.2	12	INTRONIC	0	CACNA1C: alpha 1C subunit of L-type voltage-gated calcium channel	Mediates the entry of calcium ions into cells; involved in variety of calcium-dependent processes	Calcium-depend. processes; brain volume, memory; brain disorders; CVD
rs9517320	30.8	13	INTRONIC	0	STK24: serine/threonine kinase 24	Participates in the mitogen-activated protein kinase (MAPK) cascade	Brain regeneration; memory; possibly PD and cancer
rs4148544	24.6	13	INTRONIC	0	ABCC4: ATP-binding cassette transporters family	Transport various molecules across extra- and intra-cellular membranes	Transport of xenobiotics, detoxification; cancer
rs41383	25.0	16	INTRONIC	0	NLRC5: NLR family, CARD domain containing 5	IFN-gamma-inducible nuclear transcriptional regulator of the NF-kappaB and type I interferon signaling	Innate immunity; inflammation; viral infection
rs16975963	46.7	19	NON CODING	0	AC016582.2		
rs2024714	26.8	20	INTRONIC	0	CDH4: R-cadherin (retinal)	Participates in calcium-dependent cell-cell adhesion	Cell adhesion; brain volume, brain aging; cancer
rs2826891	36.50	21	INTRONIC	0	NCAM2: neural cell adhesion molecule 2	Brain protein, superfamily of the immunoglobulin; one of plasma membrane-anchored proteins	Neural cell adhesion; cancer, brain disorders (?)
rs139170	34.7	22	INTRONIC	0	PARVG: parvin, gamma	Actin-binding proteins associated with focal adhesion	Cell adhesion; tumor suppression
rs5771675	30.9	22	INTRONIC	0	FAM19A5: member of TAFA family	Related to MIP-1alpha, a member of the CC-chemokine family.	Regulation of brain inflammation and neuronal survival (?)
rs9616906	22.7	22	UP-STREAM	−3552	AC000050.2		
rs13053175	28.0	22	UP-STREAM	−7992	RAC2: ras-related C3 botul. toxin substrate 2	GTPase of the RAS superfamily regulating cell growth, cytoskelet, and the protein kinases activation	Cell growth/ proliferation; cell adhesion; immunity, inflammation

Expanded table with references is provided in Yashin et al. (2012c).

Note: Abbreviations: AD, Alzheimer's disease; CVD, cardiovascular diseases; PD, Parkinson's disease. MAF, minor (pro-survival) allele frequency % at baseline ages (60–75).

### Empirical analyses

First, we calculated empirical estimates of the mean values of four physiological indices (BMI, DBP, SCH, and VR) in age groups <35, 35–39, …., 85–89, and 90+ years for all participants of the Original FHS cohort (males and females combined) using pooled data on measurements from all exams.

Second, we selected groups of short-lived individuals (those dying at ages 75 or earlier; censored individuals are excluded from this group) and 100 longest lived individuals (which is equivalent to individuals with lifespan exceeding 97.44 years) and evaluated average values of physiological indices in the same age groups for these individuals using pooled data on measurements from all exams.

Third, we used data on the first occurrence of CVD and cancer from the follow-up data, and data on current diabetes status from the exams to calculate ages at onset of “unhealthy life” defined as the minimum of ages of occurrence of these diseases. Then we evaluated average age trajectories of physiological indices in the same age groups described above for “unhealthy” (those with cancer, CVD, or diabetes) and “healthy” (those free of these three diseases) individuals. Measurements of physiological indices before the onset of any of these diseases contributed to the “healthy” trajectory and those after the onset of any of the diseases contributed to the “unhealthy” trajectory. Note that average values based on less than 10 observations are not shown in all figures.

Fourth, we evaluated average age trajectories in the same age groups for carriers of different numbers of alleles among the 27 pro-survival alleles selected as described in Yashin et al. (2012c). Note that the identity of SNPs was not important in this selection procedure. We calculated the total number of pro-survival alleles in the genomes of individuals using data on 27 SNPs selected in Yashin et al. (2012c). For each individual and for each of these 27 SNPs, we created a dichotomous variable equaling 1 if the individual has respective minor allele and 0 if he/she is a carrier of major allele homozygote for respective SNP. Then we created a variable counting the number of 1's for each individual (that is, the number of such minor alleles in these 27 SNPs, which could be any number from 0 to 27). The genotyped sample was divided into two sub-groups, the first containing individuals having less than 14 minor alleles and the second consisting of those having 14 or more minor alleles. For the sake of convenience, we will refer to these sub-groups as the (<14)- and the (≥14) groups, respectively. Then the age trajectories of average values of four physiological indices were calculated for individuals in these two sub-groups. Note that all analyses related to these alleles are based on data from 1471 genotyped participants.

Fifth, we estimated average trajectories among “unhealthy” and “healthy” carriers of different numbers of pro-survival alleles, as described above.

Sixth, we compared average trajectories among carriers and non-carriers of the APOE e4 allele. These calculations are based on data on APOE polymorphisms available for 1258 participants of the original FHS cohort.

Finally, we evaluated associations of the “genetic dose” with mortality rates by cause in the Cox proportional hazards model. “Genetic dose” or “polygenic score” is defined here as a variable calculating the number of pro-survival alleles [out of the 27 alleles from Yashin et al. (2012c)] in the genomes of 1471 genotyped individuals from the Original FHS cohort. The model was adjusted for sex, birth cohort, and smoking status (ever/never smoked). Age at the biospecimen collection available for the genotyped individuals was used as the left truncation in the Cox model. The model was applied to data on total mortality as well as mortality by cause (cancer, CVD, and all other or unknown causes, denoted as “Other” in Table 3).

All calculations mentioned above have been performed using SAS 9.3. Graphical output was prepared in MATLAB R2012a.

Table 2 shows subject characteristics in the specific analyses described above.

**Table 2:** **Characteristics of study subjects**.

**Study sample**	**Baseline age**				**% Females**	**Sample size**
	**Mean**	**St. Dev.**	**Min**	**Max**		
**TOTAL SAMPLE**						
	44.1	8.60	28	62	55.2%	5209
**SHORT- VS. LONG-LIVED**						
Short-lived	44.0	8.35	28	62	42.4%	1801
Long-lived	47.8	6.58	37	62	85.0%	100
**“UNHEALTHY” VS. “HEALTHY”**						
“unhealthy”	44.1	8.61	28	62	52.7%	4291
“healthy”	44.2	8.55	29	62	66.4%	788
**CARRIERS OF DIFFERENT NUMBER OF PRO-SURVIVAL ALLELES**						
<14	42.0	7.32	30	61	54.4%	721
≥14	36.0	4.50	29	55	65.7%	750
**CARRIERS (“E4”) AND NON-CARRIERS (“NOT E4”) OF THE APOE E4 ALLELE**						
e4	37.9	5.96	28	57	66.1%	277
not e4	37.6	5.66	29	57	63.1%	981
**“UNHEALTHY” AMONG CARRIERS OF DIFFERENT NUMBER OF PRO-SURVIVAL ALLELES**						
“unhealthy”, <14	41.8	7.27	30	61	52.3%	637
“unhealthy”, ≥14	35.9	4.46	29	54	63.4%	658

Notes: Short-lived are those dying at ages below 75, long-lived are those with 100 longest life spans; see details in the text. “Unhealthy” are those who had onset of at least one of the three diseases (cancer, CVD, diabetes) during the follow-up period; “healthy” are those free of such disease during the follow-up period; see more details on calculations of average trajectories in “unhealthy” and “healthy” individuals in the text. Carriers of different numbers of pro-survival alleles are defined based on the 27 SNPs identified in Yashin et al. (2012c); see details on the selection procedure in the text.

### Advanced statistical analyses using the stochastic process model

We illustrated how different aging-related characteristics in carriers of different numbers of pro-survival alleles may jointly contribute to the patterns of mortality rates as well as the trajectories of physiological variables applying the stochastic process model of aging (Yashin et al., 2007a) to data on four physiological indices (BMI, DBP, SCH, and VR) and total mortality in the 1471 genotyped individuals from the original FHS cohort. Note that data on genotyped and non-genotyped individuals can be analyzed jointly in the version of the stochastic process model described in Arbeev et al. (2009) but such analyses are beyond the scope of this paper. Some concepts and ideas about the process of aging that are used in the stochastic process model are briefly outlined in section “The need for comprehensive integrative analyses of longitudinal data.” Technical details about the specific version of the model used in this paper are given below.

#### Stochastic dynamics of individual age trajectories of physiological indices

The version of stochastic process model (Yashin et al., 2007a, 2012a), can be used to provide information about adaptive mechanisms forming the age trajectories of average physiological indices. In this model the individual dynamics of one physiological index is described by stochastic differential equation

(1) $$dY_{t}=a(t)\left(Y_{t}-f_{1}(t)\right)dt+B(t)dW_{t},Y_{0}$$

Here *Y*_*t*_ is the value of a particular physiological index at age *t* in an arbitrarily chosen individual. In contrast to traditional approaches used for analyzing longitudinal data with health or survival outcomes the description of the data in our model includes additional unobserved variables having important biological meaning for the aging process. The coefficient *B*(*t*) characterizes the contribution of random external disturbances described by a Wiener process, *W*_*t*_. Function *f*_1_(*t*) describes effect of allostatic adaptation, i.e., integrated effect of persistent external or internal disturbances which *Y*_*t*_ is forced to follow by homeostatic forces. Taking this effect into account is especially important in analyses of longitudinal data on aging, health and longevity in which measurements of external disturbances are absent or limited. This adaptation aims at achieving stability of key biological variables (not described here), through physiological or behavioral change. The strength of homeostatic forces is characterized by the negative feedback coefficient, *a*(*t*). According to (1) the age trajectory of physiological indices *Y*_*t*_ tends to follow function *f*_1_(*t*), i.e., adapt to changes in *f*_1_(*t*). An ability to adapt depends on the absolute values of *a*(*t*). Age-related changes in these coefficients characterize changes in adaptive capacity with age. Specifically, *a*(*t*) regulates the age trajectory of the physiological index approximated by *Y*_*t*_, i.e., it characterizes the rate of the adaptive response for any deviation of a physiological index from the state *f*_1_(*t*) which an organism tends to follow. For example, in a simplified one-dimensional case, when *B*(*t*) = 0, for all *t*, in Equation (1), and constant negative *a*(*t*) = *a* for all *t*, the parameter *a* is the coefficient of negative feedback in the equation for *Y*_*t*_, which keeps the trajectory *Y*_*t*_ close to *f*_1_(*t*). When *f*_1_(*t*) = *f*_1_, constant for all *t*, the value of *Y*_*t*_ asymptotically approaches *f*_1_. In case of non-zero disturbances, the higher the absolute value of *a*, the closer *Y*_*t*_ is to *f*_1_, and the faster *Y*_*t*_ tends to *f*_1_. That is why the value *a*(*t*) characterizes adaptive capacity. Practical estimation of the changes in adaptive capacity with age involves maximization of the likelihood function of the data in which *a*(*t*) is described as parametric functions of age. The random variable *Y*_0_ describes the initial value of physiological index, which is assumed to be independent of *W*_*t*_ for each *t* ≥ 0. The introduction of *f*_1_(*t*) and *a*(*t*) into the model facilitates the biological interpretation of the results of statistical analyses of longitudinal data.

#### Conditional risk function (conditional mortality rate)

Note that individual trajectories of physiological variables must be stopped at random time *T* describing lifespan of an individual. The probability distribution of this stopping time is characterized by conditional mortality rate given the value of the physiological index. This conditional mortality rate is represented by the quadratic form:

(2) $$\mu \left(t,Y_{t}\right)=\mu_{0}(t)+Q(t){\left(Y_{t}-f_{0}(t)\right)}^{2}$$

The term μ_0_(*t*) (the baseline mortality rate) is a function of age. It shows how the total mortality rate would change if a corresponding physiological index *Y*_*t*_ followed the optimal trajectory *f*_0_(*t*). The function *f*_0_(*t*) is associated with the notion of the age-dependent “norm” in the model. The positive function *Q*(*t*) shows how the steepness of the parabola *Q*(*t*) (*Y*_*t*_ − *f*_0_(*t*))^2^ changes with increasing age.

The model described above takes into account the fact that available longitudinal data do not contain records characterizing when, how, and how long external disturbances affected individuals during their life course. The use of the notions of allostasis and allostatic adaptation helps us understand how persistent unfavorable conditions get “under the skin” of affected person, increasing his/her susceptibility to diseases and death (McEwen, 2012). Many such conditions affect set-points of physiological homeostasis changing physiological balance from the “normal,” *f*_0_(*t*) to “abnormal,” *f*_1_(*t*) ≠ *f*_0_(*t*) state. These effects, represented in Equation (1) can be estimated from the FHS data thereby providing indirect evaluation of the effects of external disturbances without measuring them.

#### Version of model used in application to FHS data

We applied a discrete-time version (see Yashin et al., 2007b) of the general model (1)–(2) (with values of a physiological index evaluated at one-year age intervals using respective observations in the adjacent FHS exams) with specification of respective components as follows. We used a constant diffusion *B*(*t*) = σ_1_; a linear function for the adaptive capacity *a*(*t*): *a*(*t*) = *a*_*Y*_ + *b*_*Y*_*t*; a linear function for the quadratic hazard term *Q*(*t*): *Q*(*t*) = *a*_*Q*_ + *b*_Q_*t*, and the Gompertz function for the baseline hazard μ_0_(*t*):μ_0_(*t*) = *a*_μ_0__exp(*b*_μ_0__*t*). Initial values *Y*_0_ are assumed normally distributed, *N*(*f*_1_(*t*_0_), σ_0_).

To evaluate the “optimal” trajectories *f*_0_(*t*), we calculated the average age trajectories of physiological variables for long-lived individuals (those with lifespan exceeding 90 years) in the original FHS cohort and fitted these trajectories by cubic polynomials using the Curve Fitting Toolbox in MATLAB. The fitted curves were used as the estimates of the “optimal” trajectories *f*_0_(*t*) [see motivation for such specification in Yashin et al. (2012b)]. Note that longitudinal observations in long-lived individuals are available only starting at ages 40 and above. Therefore, we restricted applications of our model to observations at ages 40 and above.

Taking into account the possibility that the homeostatic regulation forces the trajectory of physiological variables to the values different from the optimal values represented by *f*_0_(*t*) and that this difference can be age-dependent, we specified the function *f*_1_(*t*) as *f*_1_(*t*) = *f*_0_(*t*) + Δ *f*_0_(*t*), where Δ*f*_0_(*t*) = *a*_*f*_0__ + *b*_*f*_0__*t*.

Details of the likelihood maximization procedure can be found in Yashin et al. (2007a,b). The likelihood maximization was performed using the constrained optimization procedure of MATLAB's Optimization Toolbox. The constrained maximization algorithm was used to impose necessary restrictions on parameters of: (1) function *f*_1_(*t*), to ensure “physiologically reasonable” values at each age; (2) the feedback coefficient *a*(*t*), to ensure its negative value at each age so that the trajectories of *Y*_*t*_ tend to *f*_1_(*t*); (3) the baseline hazard μ_0_(*t*), to ensure non-negative values for each age; (4) σ_0_, and σ_1_, to ensure non-negative values; and (5) the quadratic hazard term *Q*(*t*), to ensure that the values are non-negative for each age.

The model was applied to data on the two groups of genotyped individuals from the original FHS cohort, those carrying <14 and ≥14 alleles out of the 27 pro-survival alleles selected in Yashin et al. (2012c). First, we estimated the unrestricted model that has all different parameters in the two groups and then restricted models imposing respective restrictions on the parameters of the model to test the null hypotheses about the equality of model's characteristics in the two groups. Specifically, we tested four null hypotheses on the equality of (1) baseline hazards, (2) quadratic hazard terms, (3) adaptive capacities, and (4) mean allostatic trajectories in the two groups. The hypotheses were tested using the likelihood ratio test. Respective *p*-values are shown in Figures 9–11.

## Results

### The non-monotonic average age trajectories of physiological variables

**Figure 2:**
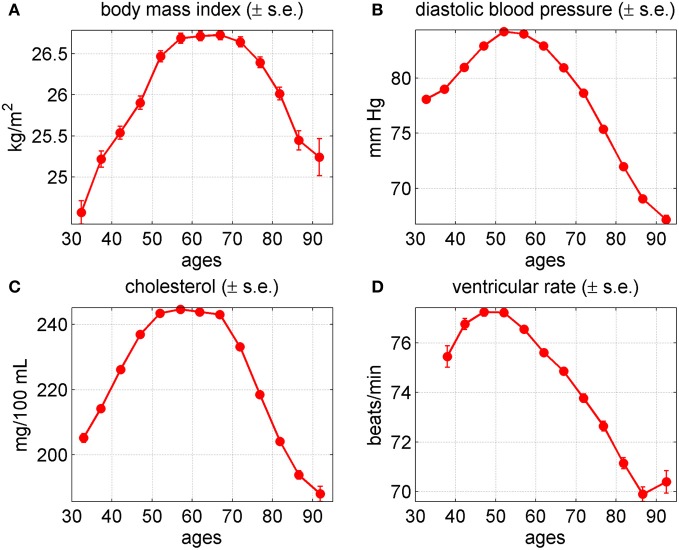
**Average trajectories (±standard errors) of four physiological variables in the Framingham Heart Study (original cohort, pooled data from exams 1–28). (A)** Body mass index; **(B)** diastolic blood pressure; **(C)** cholesterol; **(D)** ventricular rate.

The shapes of the average age trajectories of physiological indices (Figure 2) provide useful insights about factors and mechanisms involved in changes developing in aging human body which can be verified using more sophisticated statistical approaches. Figure 2 displays the age patterns of average values of physiological indices for BMI, DBP, SCH, and VR for males and females combined, evaluated from the data on the Original FHS cohort.

One can see from this figure that all four trajectories are non-monotonic. After a period of increase at ages between 35 and 50–54 years, the values of BMI, DBP, SCH, and VR reached their maximum value and then declined. Note that the shapes of these curves do not necessarily represent the shapes of biological age trajectories in individual organisms. This is because in addition to the contribution of biological aging, these trajectories reflect the effects of compositional changes due to mortality selection in a heterogeneous population.

The average age patterns for males and females look similar to those of the combined estimates, with some differences in details (not shown). Specifically, the average values of BMI for females were lower than those of males at ages between 35 and 74 years. They increased faster and reached their maximum value later than those of males. After age 75 the values of BMI coincided for the two genders. The average values of DBP for females were lower than those of males at ages between 35 and 59. They increased faster and reached their maximum value later than those of males. After age 60, the values of DBP practically coincided with those of males with a tendency to become higher at age 95 years. The average values of SCH for females were lower than those of males at ages between 35 and 44 years. Then they became higher than those of males for the rest of the age domain. The values of SCH for females increased faster and reached their maximum value later than those of males. The values of VR for females were higher than those of males at the entire age domain.

### The age trajectories of the short lived (SL) vs. the longest lived (LL) individuals

**Figure 3:**
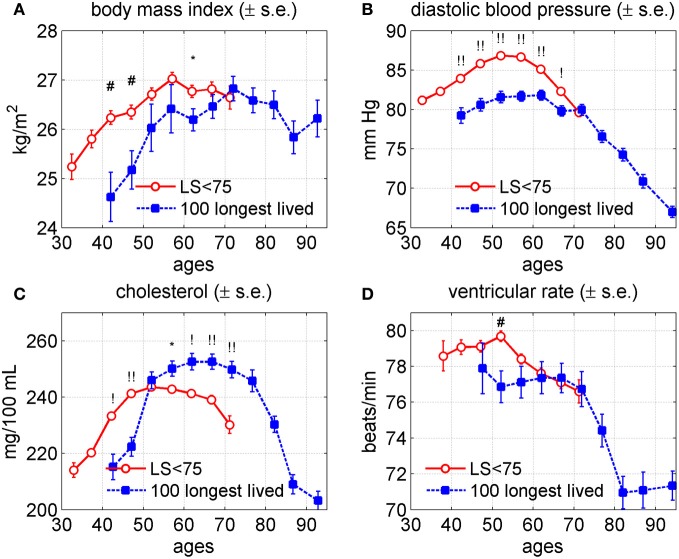
**Average trajectories (±standard errors) of four physiological variables in groups of short-lived individuals (“LS < 75,” i.e., those dying at ages 75 or earlier; censored individuals are excluded from this group) and 100 longest lived individuals in the Framingham Heart Study (original cohort, pooled data from exams 1–28). (A)** Body mass index; **(B)** diastolic blood pressure; **(C)** cholesterol; **(D)** ventricular rate. Symbols above the curves correspond to *p*-values for testing the null hypotheses on equality of means in respective age groups: no symbol: *p* ≥ 0.05; ^*^: 0.01 ≤ *p* < 0.05; ^#^: 0.001 ≤ *p* < 0.01; ^!^: 0.0001 ≤ *p* < 0.001; ^!!^: *p* < 0.0001.

Figure 3 shows the average age trajectories of DBP, BMI, SCH, and VR, for the short lived (lifespan, LS <75 years) and the 100 longest lived (aged 97+) males and females. One can see that trajectories for the LL individuals were substantially different from those for the SL individuals in all four indices. Specifically, the average values of BMI were higher among the SL persons until age 70 with tendency to intersect trajectory of this index for the LL individuals. The age trajectories of BMI for the SL and LL groups reached their maximum values at ages 60 and 70, respectively. The average values of DBP among the SL people were higher than those of the LL study participants from age 40 until 70 years of age. At ages 70–74 years the values of DBP were practically indistinguishable between the two groups. The maximum value of the average DBP in the SL group was higher than that of the LL persons and it was reached earlier (50 years for the SL and about 60 years for the LL individuals). The average values of SCH were higher among the SL persons up to age 55, where they reached their maximum value and then declined. At age 55 the average age trajectory of SCH for the SL persons intersected that of the LL persons. The average age trajectory of the SCH for the LL persons reached their maximum value about age 65 and then declined. Note that at average, after age 55 females have higher levels of SCH than males. This fact together with information that females comprise 85% of the LL group and only 57.6% of the SL group (Table 2) contributes to difference in magnitudes between SCH trajectories for the SL and LL individuals in Figure 3. The values of VR for the SL persons were higher until age 65 and then practically coincided with that of the LL people until age 75 with the tendency to intersect.

**Figure 4:**
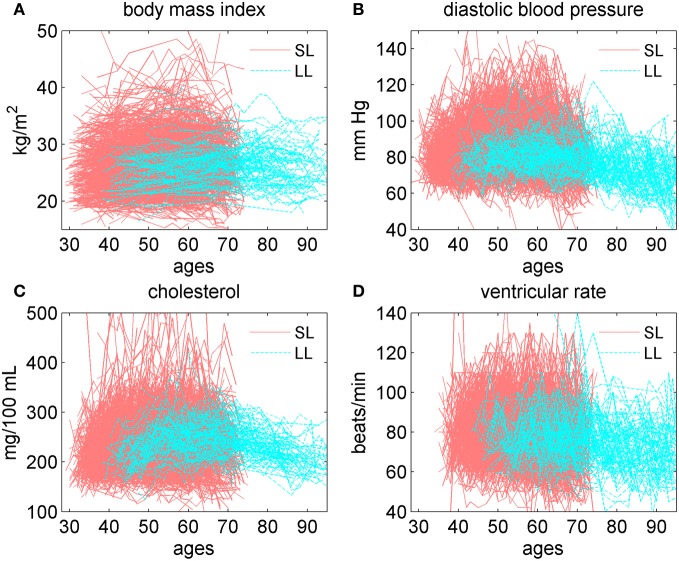
**Individual trajectories of four physiological variables in groups of short-lived individuals (“SL,” those dying at ages 75 or earlier; censored individuals are excluded from this group) and 100 longest lived (“LL”) individuals in the Framingham Heart Study (original cohort, pooled data from exams 1–28). (A)** Body mass index; **(B)** diastolic blood pressure; **(C)** cholesterol; **(D)** ventricular rate.

Figure 4 displays individual trajectories for the SL and LL groups. It reveals that there is some tendency in trajectories of the LL individuals (to a lesser extent in VR) to avoid extreme values of indices (which, however, may be just an artifact of a smaller number of individuals in the LL group). Nevertheless, Figure 4 generally shows that, despite the observed differences in average patterns (Figure 3), different individuals may have very diverse trajectories and the SL individuals are a more heterogeneous group.

### The age trajectories of physiological indices for healthy and unhealthy individuals

**Figure 5:**
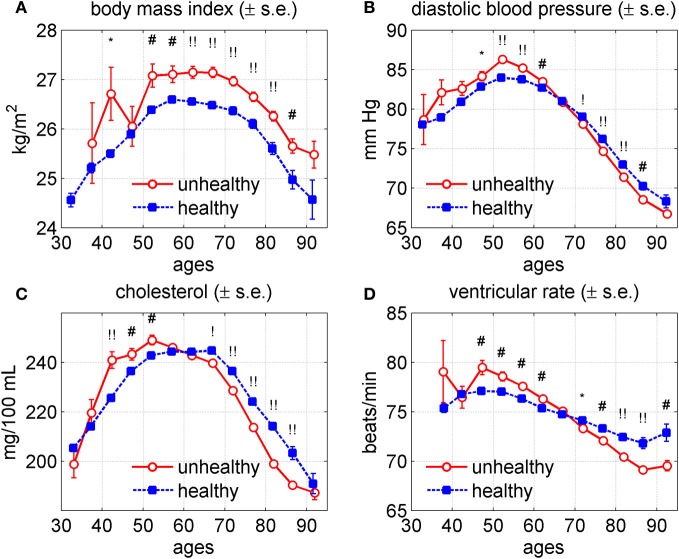
**Average trajectories (±standard errors) of four physiological variables for “unhealthy” and “healthy” individuals in the Framingham Heart Study (original cohort, pooled data from exams 1–28). (A)** Body mass index; **(B)** diastolic blood pressure; **(C)** cholesterol; **(D)** ventricular rate. Note: “unhealthy” individuals are those with cancer, CVD or diabetes; “healthy” are those free of these three diseases. Measurements of physiological indices before the onset of any of these diseases contribute to the “healthy” trajectory and those after the onset of any of the diseases contribute to the “unhealthy” trajectory. Symbols above the curves correspond to *p*-values for testing the null hypotheses on equality of means in respective age groups: no symbol: *p* ≥ 0.05; ^*^: 0.01 ≤ *p* < 0.05; ^#^: 0.001 ≤ *p* < 0.01; ^!^: 0.0001 ≤ *p* < 0.001; ^!!^: *p* < 0.0001.

It is well known from medical practice that health status may influence values of physiological indices, as well as lifespan. In turn, physiological variables associated with lifespan are likely to show associations with some chronic diseases. To elucidate links between health and physiological variables we calculated average age trajectories of BMI, DBP, SCH, and VR for healthy and unhealthy individuals. The unhealthy individuals are defined here as those having at least one of three diseases: cancer, CVD, or diabetes. Since having a disease is likely to increase mortality risk, the unhealthy individuals are likely to be more susceptible to death. Figure 5 shows average age trajectories for healthy and unhealthy persons (males and females combined).

One can see from this figure that the trajectories differed for healthy and unhealthy individuals. Specifically, the average values of BMI tended to be higher for the unhealthy than for healthy group at ages from 40 to 95 years. The average trajectories of DBP, SCH, and VR for healthy and unhealthy persons intersected at ages between 60 and 70 years. The values of SCH were higher among unhealthy individuals at age between 40 and 60 years. Then the curves intersected, so after age 60 the healthy individuals had higher values of SCH. The values of DBP had a similar pattern with the intersection point around age 65, which was, however, less pronounced. The values of VR were higher among unhealthy people until about age 65, after which they became lower than those among healthy people.

### Genetic influence on age trajectories of physiological indices

**Figure 6:**
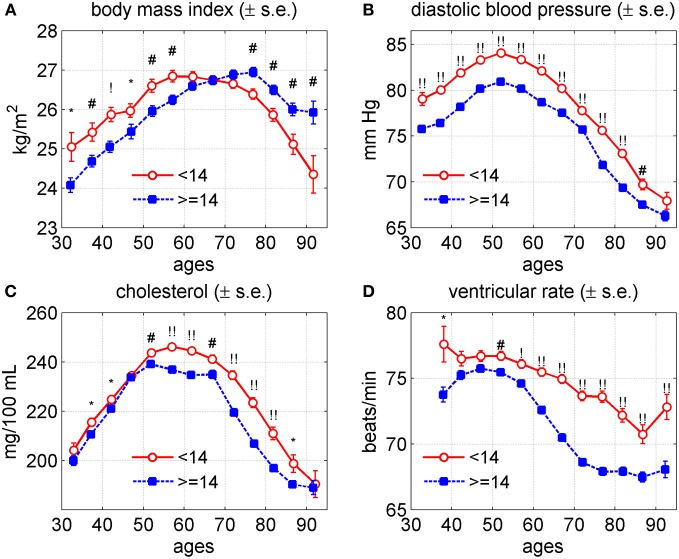
**Average trajectories (±standard errors) of four physiological variables in the Framingham Heart Study (original cohort, pooled data from exams 1–28) for individuals carrying different number of pro-survival alleles (<14 and ≥14) out of the 27 such alleles identified in Yashin et al. (2012c). (A)** Body mass index; **(B)** diastolic blood pressure; **(C)** cholesterol; **(D)** ventricular rate. Symbols above the curves correspond to *p*-values for testing the null hypotheses on equality of means in respective age groups: no symbol: *p* ≥ 0.05; ^*^: 0.01 ≤ *p* < 0.05; ^#^: 0.001 ≤ *p* < 0.01; ^!^: 0.0001 ≤ *p* < 0.001; ^!!^: *p* < 0.0001.

In Yashin et al. (2012c), we showed that human lifespan in the Original FHS cohort was positively associated with the “dose” of 27 individually selected genetic variants (“longevity” alleles) each having a small positive effect on lifespan. It is clear that genetic effects on lifespan and survival are mediated by a number of intermediate variables whose effects are integrated in the values of physiological variables. Since the genetic dose index was associated with lifespan and the values of physiological variables measured in the Original FHS cohort were also associated with lifespan we expected that the genetic dose index would show an association with the age trajectories of the physiological variables affecting lifespan as well. To illustrate this, we divided the genotyped population of study participants into two sub-cohorts. The first one included individuals carrying up to 13 out of 27 alleles associated with lifespan in our earlier study. Individuals from the second sub-cohort carry 14 and more such alleles in their genomes, referred as the (<14)- and the (≥14)-groups, respectively. Figure 6 shows how the age trajectories of BMI, DBP, SCH, and VR differed between the (<14)- and the (≥14)-groups of study participants for the two genders combined.

One can see from this figure that the average age trajectories of BMI for the (<14)-group were higher up to age 65 years and then became lower than those in the (≥14)-group. Note that the maximum value of BMI for the members of (<14)-group was reached earlier (around 55 years of age) than for the members of the (≥14)-group (around 75 years). The individuals from the (<14)-group had higher levels of the average values of DBP and VR for the entire age interval 35–95 years shown in the graphs. The average values of the SCH for this group were higher than those for the (≥14)-group between ages 50 and 95 years and were about the same beyond this interval.

The average age trajectories of these physiological indices varied slightly between males and females (figures not shown). Specifically, the average values of BMI among female members of the (<14)-group were higher until age 70, stayed about the same until age 85, and intersected those of the group (≥14) after this age. In males, the values of this variable tended to be higher in the (<14)-group until age 60, became lower than those in the (≥14)-group and then became about the same at age 85 years. For DBP the pattern of differences between the two groups remained the same for each of the two genders with larger differences in DBP trajectories between the groups in females than in males. In males, the values of VR in the two groups were about the same until age 50 years and then diverged. The female levels of SCH in the (<14)-group were higher than in the (≥14)-group until age 85 and were about the same after this age. In males, the values of SCH remained higher in this group until age 50, stayed about the same in both groups until age 75, and then they become lower for the members of the (<14)-group. For comparison we will show age patterns of the same physiological variables for carriers and non-carriers of the APOE-e4 allele.

### The age trajectories of physiological indices for carriers and non-carriers of the APOE-e4 allele

The association of the APOE alleles with longevity has been detected in many genetic studies (e.g., Deelen et al., 2011; Nebel et al., 2011). Their influences on age trajectories of physiological indices are less known. In Arbeev et al. (2012) we evaluated effects of the APOE polymorphism on age trajectories of SCH and DBP in the original FHS cohort and found differences in average age trajectories of these indices in long-lived carriers and non-carriers of the e4 allele. The analyses also showed that the average age trajectories in individuals dying at earlier ages markedly deviate from those of the long-lived groups and these patterns differ for carriers and non-carriers of the e4 allele of both sexes. Applying the extended version of the stochastic process model (Arbeev et al., 2009) we found the presence of a genetic component in aging-related mechanisms that is manifested in the observed patterns of the allele-specific age trajectories of physiological indices and mortality rates.

**Figure 7:**
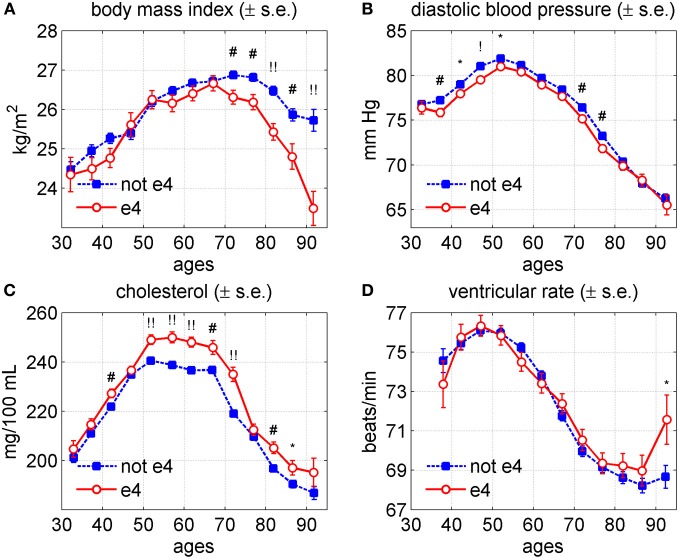
**Average trajectories (±standard errors) of four physiological variables for carriers (“e4”) and non-carriers (“not e4”) of the APOE e4 allele in the Framingham Heart Study (original cohort, pooled data from exams 1–28). (A)** Body mass index; **(B)** diastolic blood pressure; **(C)** cholesterol; **(D)** ventricular rate. Symbols above the curves correspond to *p*-values for testing the null hypotheses on equality of means in respective age groups: no symbol: *p* ≥ 0.05; ^*^: 0.01 ≤ *p* < 0.05; ^#^: 0.001 ≤ *p* < 0.01; ^!^: 0.0001 ≤ *p* < 0.001; ^!!^: *p* < 0.0001.

To compare whether the effects of the APOE alleles are similar to those of genetic variants we calculated average age trajectories of the BMI, DBP, SCH, and VR for study participants carrying and not carrying APOE-e4 allele. These trajectories are shown in Figure 7 for males and females combined.

One can see from this figure that the values of DBP and VR were about the same for carriers and non-carriers of APOE-e4. The most significant difference was in BMI. The curves look about the same until age 65, and then diverge. Among the APOE-e4 carriers, BMI starts to decline. Among non-carriers the values of BMI continued to increase until age 75 and then declined. The rate of decline was higher among carriers of the APOE-e4. The SCH levels were about the same until age 50, after which they became higher in the carriers of the APOE-e4 and remain slightly higher for the rest of the age interval. The figures for males and females are not much different from combined estimates (the graphs are not shown). The figures for females repeat those for the two sexes combined. For males, the values of DBP among APOE-e4 carriers were slightly lower than in non-carriers until age 55, stayed about the same until age 75, and then slightly exceeded those of non-carriers. Comparing Figure 7 with Figure 6 indicates that the effect of the APOE-e4 on SCH is similar to that of the (<14)-group at the entire age domain; for BMI the effect is similar after age 65; for DBP the effect is opposite but much less pronounced; and for VR the APOE-e4 effect does not exist.

### Genetics of age trajectories for healthy and unhealthy individuals

**Figure 8:**
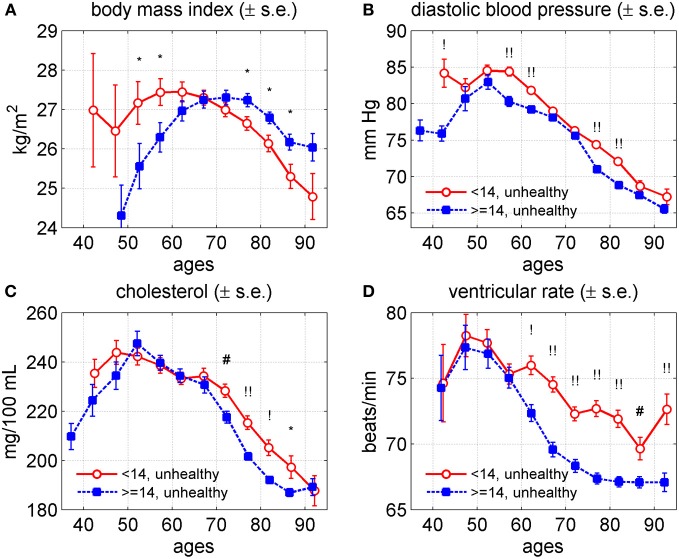
**Average trajectories (±standard errors) of four physiological variables in the Framingham Heart Study (original cohort, pooled data from exams 1–28) for “unhealthy” individuals carrying different number of pro-survival alleles (<14 and ≥14) out of the 27 such alleles identified in Yashin et al. (2012c). (A)** Body mass index; **(B)** diastolic blood pressure; **(C)** cholesterol; **(D)** ventricular rate. Note: “unhealthy” individuals are those with cancer, CVD, or diabetes; “healthy” are those free of these three diseases. Measurements of physiological indices before the onset of any of these diseases contribute to the “healthy” trajectory and those after the onset of any of the diseases contribute to the “unhealthy” trajectory. Symbols above the curves correspond to *p*-values for testing the null hypotheses on equality of means in respective age groups: no symbol: *p* ≥ 0.05; ^*^: 0.01 ≤ *p* < 0.05; ^#^: 0.001 ≤ *p* < 0.01; ^!^: 0.0001 ≤ *p* < 0.001; ^!!^: *p* < 0.0001.

The graphs of the age trajectories of BMI, DBP, SCH, and VR for unhealthy individuals of the (<14)- and (≥14)-groups are shown in Figure 8 for the two sexes combined.

The average values of BMI in the (<14)-group were higher than those in the (≥14)-group until age 65. Then the trajectories intersected so the values of BMI in the (<14)-group became smaller than those in the (≥14)-group for the rest of the age domain. The average values of DBP in both groups were about the same between ages 65 and 75 years. However, beyond this interval the values of this index in the (<14)-group were higher than those in the (≥14)-group. The average levels of SCH were about the same until age 65. Then they become higher in the (<14)-group until age 95 years. The average values of VR were about the same until age 55 and then became higher for individuals from the (<14)-group.

The graphs of the age trajectories of BMI, DBP, SCH, and VR for healthy individuals of the (<14)- and (≥14)-groups for the two sexes combined practically repeat those shown in Figure 6 for the entire population and therefore are not shown.

### The effects of genetic dose on mortality by cause

The results of application of the Cox model to cause-specific mortality data (see details in section “Empirical analyses”) are shown in Table 3. One can see from this table that the estimates of the effects on all mortality rates are statistically significant.

**Table 3:** **Effects of polygenic score on total and cause-specific mortality in the Cox model**.

**Cause**	**Events #**	**Cens. #**	**Beta**	**SE**	***p*-value**	**HR (95% C.I.)**
Total	1267	204	−0.057	0.007	2.7E-18	0.94 (0.93; 0.96)
CVD	368	1103	−0.047	0.012	0.0001	0.95 (0.93; 0.98)
Cancer	243	1228	−0.030	0.015	0.041	0.97 (0.94; 0.99)
Other	656	815	−0.071	0.009	1.9E-15	0.93 (0.92; 0.95)

Notes: “Cause” denotes the cause of death (Total—all causes, CVD, cancer, and Other—all other causes or unknown cause); “Events #” shows the number of deaths from specific cause; “Cens. #” indicates the number of censored cases; “Beta” shows the estimate of the regression coefficient for the polygenic score variable in the Cox model; “SE” is a standard error for Beta; “p-value” characterizes the significance of the estimate; “HR” shows the estimate of hazard ratio per one SNP (pro-survival allele), 95% confidence intervals are shown in parentheses.

### The need for comprehensive integrative analyses of longitudinal data

The values of physiological variables described above are regulated by dynamic biological mechanisms which integrate external influence, internal changes and compensatory activity. An essential part of such mechanism is the negative feedback loop which tends to stabilize the values of regulated variables around certain set points. Many variables involved in regulation of observed physiological indices are not observed, and therefore are not represented in the longitudinal data. To be able to address research questions about fundamental regularities of changes developing in aging human body, the behavior of these additional variables has to be investigated together with the values of physiological variables measured in the study. For these purposes we developed a version of the stochastic process model of human mortality and aging which allows for incorporating established facts, new research findings, and a number of theoretical concepts about aging into the model. Specifically, we used linear stochastic differential equations to describe aging related changes in physiological variables driven by the effect of allostatic adaptation to persistent external disturbances, stochastic components, and subjected to regulation by negative feedback mechanism, which properties may change during the life course. The dynamic effects of physiological variables on mortality risk are described by conditional hazard having quadratic form. This reflects empirical evidence of the U- or J-shapes of risks considered as functions of risk factors (e.g., observed covariates). Variables describing changes in stress resistance and adaptive capacity modulate quadratic hazard and feedback regulation mechanism, respectively. Variables describing physiological norm characterize “optimal” values of conditional risks. Additional variables characterize levels of stochasticity and effects of allostatic adaptation (Yashin et al., 2007a, 2012a).

The traditional way of describing dynamics of systems with such regulation is the use of ordinary differential equations. To take the random factors that tend to shift the values of corresponding variables from their normal set points into account, the stochastic differential equation with feedback is used (Yashin and Manton, 1997; Yashin et al., 2007a). In the ideal (no stresses) situation the set point of the feedback regulation mechanism for a given variable corresponds to its normal value for a given age, i.e., the value that minimizes mortality risk at this age. In the presence of persistent external disturbances the regulation set point deviates from its normal value by the process of allostatic adaptation. The absolute value of difference between the normal and the realized set points is called allostatic load. The allostatic load may change with age reflecting continuing adjustment of an organism to persistent environmental conditions. Note that neither these conditions nor allostatic load are observed, although a number of studies developed a proxy measure of these characteristics using available measurements (McEwen and Seeman, 1999; McEwen, 2000, 2012). In our analyses the variable representing allostatic load can be introduced into the model and estimated indirectly from longitudinal data. The quality of feedback regulation is determined by the absolute value of the feedback coefficient. This value characterizes the “adaptive capacity” of a system. Individuals with better capacity are likely to be healthier and have longer life.

#### Two mechanisms regulating average values of the physiological index in the cohort

It is shown by Yashin et al. (2007a) that average trajectories of any physiological index (*m*(*t*)) described by Equations (1) and (2) satisfy the following ordinary differential equation:

(3) $$dm(t)/dt=a(t)\left(m(t)-f_{1}(t)\right)-2\gamma (t)Q(t)(m(t)-f_{0}(t)),m(0)=m_{0}$$

Here the coefficient γ(*t*) is a positive function of age (Yashin et al., 2007a), and all other coefficients are specified in the descriptions of Equations (1) and (2). The coefficient *a*(*t*) is negative by the definition. The coefficient *Q*(*t*) is non-negative by definition. Thus equation (3) describes two negative feedback mechanisms regulating age trajectory of *m*(*t*). One of them, characterized by the feedback loop coefficient *a*(*t*), deals with homeostatic adaptation to the values of slow-time allostatic response *f*_1_(*t*) to persistent external disturbances (e.g., stresses of life). This mechanism tries to keep the value of *m*(*t*) around function *f*_1_(*t*). The second mechanism is represented by the negative feedback loop describing the effect of mortality selection on *m*(*t*) (average age trajectory of physiological index). This mechanism is characterized by the feedback coefficient −2γ(*t*)*Q*(*t*). Its task is to keep the value of *m*(*t*) around function *f*_0_(*t*), the optimal age trajectory of physiological index, (i.e., the function of age which minimizes the mortality risk at each given age).

#### Application to data

**Figure 9:**
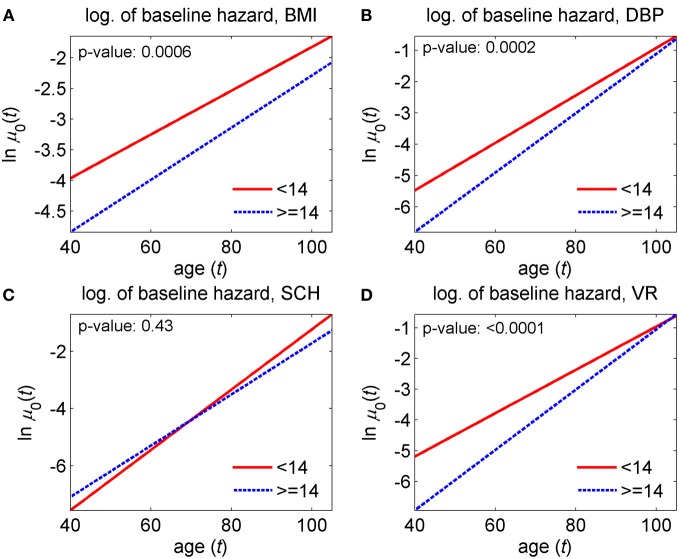
**Estimates of the logarithm of the baseline hazard rates in the stochastic process model (Yashin et al., 2007a) applied to data on longitudinal measurements of four physiological indices and total mortality in individuals carrying different number of pro-survival alleles (<14 and ≥14) out of the 27 such alleles identified in Yashin et al. (2012c). (A)** Estimates for body mass index (BMI); **(B)** estimates for diastolic blood pressure (DBP); **(C)** estimates for cholesterol (SCH); **(D)** estimates for ventricular rate (VR). *P*-values are for the null hypotheses on the equality of baseline hazards in the two groups. See more details about the model in section “Advanced statistical analyses using the stochastic process model.”

**Figure 10:**
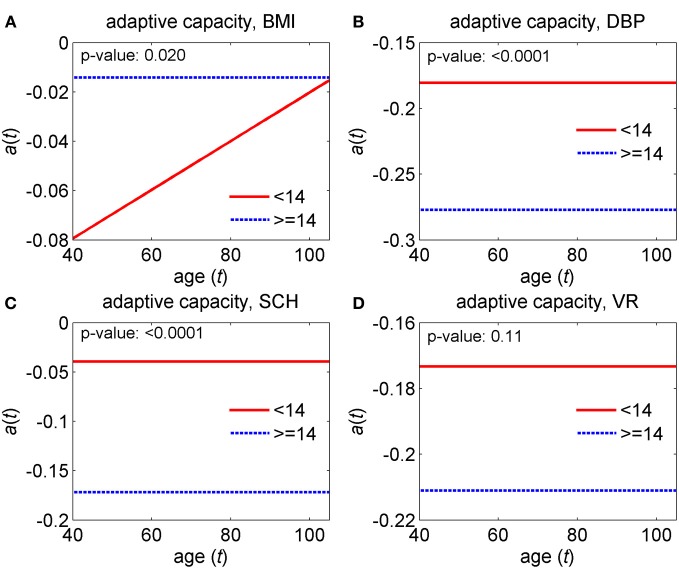
**Estimates of adaptive capacity in the stochastic process model (Yashin et al., 2007a) applied to data on longitudinal measurements of four physiological indices and total mortality in individuals carrying different number of pro-survival alleles (<14 and ≥14) out of the 27 such alleles identified in Yashin et al. (2012c). (A)** Estimates for body mass index (BMI); **(B)** estimates for diastolic blood pressure (DBP); **(C)** estimates for cholesterol (SCH); **(D)** estimates for ventricular rate (VR). *P*-values are for the null hypotheses on the equality of adaptive capacities in the two groups. See more details about the model in section “Advanced statistical analyses using the stochastic process model.”

**Figure 11:**
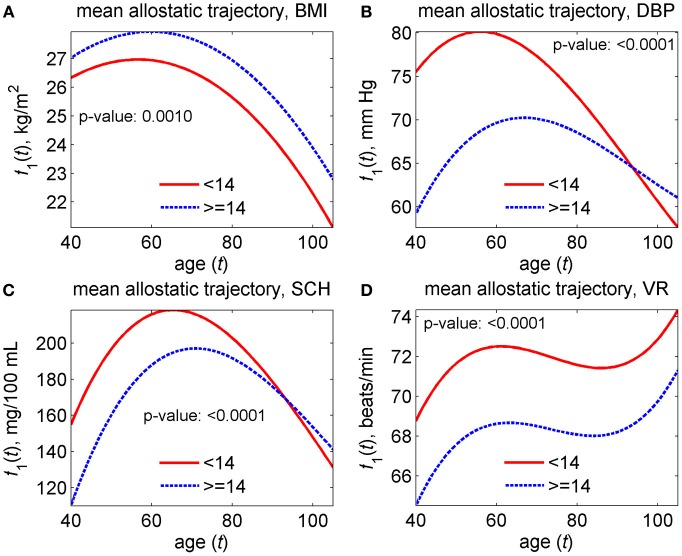
**Estimates of mean allostatic trajectories in the stochastic process model (Yashin et al., 2007a) applied to data on longitudinal measurements of four physiological indices and total mortality in individuals carrying different number of pro-survival alleles (<14 and ≥14) out of the 27 such alleles identified in Yashin et al. (2012c). (A)** Estimates for body mass index (BMI); **(B)** estimates for diastolic blood pressure (DBP); **(C)** estimates for cholesterol (SCH); **(D)** estimates for ventricular rate (VR). *P*-values are for the null hypotheses on the equality of mean allostatic trajectories in the two groups. See more details about the model in section “Advanced statistical analyses using the stochastic process model.”

The version of this general model has been used in the analyses of data on four physiological indices (BMI, DBP, SCH, and VR) collected in the Original FHS cohort (see section “Advanced statistical analyses using the stochastic process model”). The results of these analyses of longitudinal data are shown in Figures 9–11. One can see from these figures that parameters of corresponding models are successfully estimated, and age trajectories of corresponding variables can be well interpreted. Substantially, the analyses revealed significant differences in the parameters of the model associated to the baseline hazard rates (except SCH), the adaptive capacities (except VR) and the average allostatic trajectories between the two sub-groups of individuals carrying <14 and ≥14 pro-survival alleles.

Figure 9 shows that the baseline hazard (i.e., the hazard summarizing the effect of all factors except respective physiological variable) is lower in carriers of a larger number of pro-survival alleles (≥14), compared to carriers of a smaller number of such alleles (<14) for all variables except SCH.

The lack of significant differences in the baseline hazard rates for SCH indicates that the differences in survival chances in the two groups can be explained by differences in other characteristics such as adaptive capacities and average allostatic trajectories (see Figures 10C and 11C). Note also that for two indices, DBP and VR, the initial value of the baseline hazard is smaller but its slope is larger in carriers of a larger number of alleles (≥14) and the curves intersect at some advanced age (around 105 years). This means rectangularization of respective “baseline” survival functions (when going from the (<14)- to the (≥14)-group). We note here that the results for BMI should be interpreted with care because the quadratic term for the (<14)-group estimated as zero for this index (data not shown).

Figure 10 displays the estimates of the feedback coefficient representing adaptive capacity in the model.

It reveals that for two physiological variables, DBP and SCH, carriers of a larger number of pro-survival alleles have significantly better adaptive capacity [i.e., larger absolute values of the feedback coefficient in Equation (1)] than individuals with a smaller number of such alleles. That is, in the group carrying a larger number of pro-survival alleles, the trajectories of these physiological variables return faster to the average “allostatic” trajectories that organisms are forced to follow than they do in the individuals carrying a smaller number of pro-survival alleles.

Figure 11 shows these average trajectories that the organisms are forced to follow by the process of allostatic adaptation (“mean allostatic trajectories”) in the two groups.

One can see from this figure that the processes of allostatic adaptation in individuals carrying different number of pro-survival alleles act differently in the sense that the resulting trajectories are significantly different in the two groups. The pattern differs by physiological variables. Specifically, the trajectory for the (<14)-group is consistently lower for BMI, consistently higher for VR, and shows similar patterns for the other two variables (DBP and SCH): for the (<14)-group the trajectory is initially higher, reaches the maximum and starts declining at earlier ages, and declines at a faster rate compared to the (≥14)-group so that the trajectories in the two groups intersect at ages about 90–95. Finally, we observed that the (≥14)-group has a decline in the quadratic hazard term *Q*(*t*) with age which means a widening [not narrowing as in the (<14)-group] U-shape of the mortality risk as a function of a physiological variable with age. However, no significant conclusion can be made about the difference in behavior of the quadratic hazard term in these two groups for all physiological variables because of non-significant *p*-values (about 0.4) and we do not show the results here.

In sum, the evaluated hidden mechanisms of aging-related changes can collectively explain the difference in the mortality risk in the two groups carrying different number of pro-survival alleles. Note that these analyses illustrate the effect of only specific longitudinal variables. As the significant differences between baseline hazard rates in the two groups reveal, there can be many more factors that can explain the difference in the mortality rates in these groups.

## Discussion

### Extreme longevity and health are linked to distinct types of changes in age-trajectories of physiological indices

Figures 3 and 5 indicate that exceptional longevity and health may be linked to distinct types of changes in the age-trajectories of physiological indices. Indeed, a common point of the differences between the longest versus shorter living individuals, as seen in Figure 3, is that for the LL individuals *the value of an index peaked later in life and started to decline about 10 years later* as compared with the SL individuals. For BMI, in particular, the patterns of its aging changes had similar shape for the LL and SL, as well as had similar peak values of the index. The major difference was that for the LL individuals, the whole age-pattern of the BMI was shifted to the right, and all BMI changes were postponed in time.

This indicates that a postponement of physiological aging changes in time may be essential for achieving extreme longevity. It seems logical, especially if to look at strains of lab animals which significantly differ in longevity and are thought to age at a different pace [such as B6 vs. DBA mice, e.g., Sell and Monnier (1997)]. The longer compared to shorter living strains typically have all the stages of aging changes in physiological indices (such as the rise, peak or decline in BMI) shifted toward an older age (Turturro et al., 1999).

While the typical difference between the long and short living individuals was the shift of the age-trajectory of physiological changes toward older age, the respective trajectories for healthy and unhealthy individuals had similar timing and overall similar age at the peak value of the index, with probable exception for cholesterol (Figure 5).

Our results thus suggest that pathways to achieving extreme longevity and health are not necessary the same, or at least do not always overlap and may potentially involve significant trade-offs. For example, consider BMI in Figures 3 and 5. Why indeed is the average BMI in healthy old people substantially lower than that BMI in the longest living individuals for similar age intervals 75 + ? One reason could be that at older ages (and especially at the oldest old ages), the level of overall resistance to stresses (such as ability to cope with infections, fractures, bleeding, sarcopenia, atrophy, and generally frailty) starts to play a more important role in person's survival than specific diseases. This is reflected in declining the relative excess of mortality attributed to each specific cause of death with advancing age (Forsen et al., 1999; Horiuchi et al., 2003; Richmond et al., 2003). And as it was discussed at a recent International Conference on Sarcopenia Research, having an excess of fat storage in the body may potentially help to postpone frailty and improve the relative chances of survival in both healthy and unhealthy individuals at older ages (Doehner et al., 2012).

Figure 3 shows that longest lived subjects have significantly higher SCH levels, which seems to contradict with the general knowledge from epidemiological studies that higher SCH levels are associated with a higher mortality. Although the literature analyses show controversial results the evidence is accumulating that the values of total cholesterol have different effects at different age intervals. Specifically, Kronmal et al. (1993) found that the relationship between total cholesterol level and all-cause mortality was positive at age 40 years, negative at age 80 years, and negligible at ages 50–70 years. Krumholz et al. (1994) concluded that the results of their analyses do not support the hypothesis that hypercholesterolemia or low HDL-C are important risk factors for all-cause mortality, coronary heart disease mortality, or hospitalization for myocardial infarction or unstable angina in this cohort of persons older than 70 years. Weijenberg et al. (1996) reported that total cholesterol seems to be a stronger risk factor for mortality from the disease, whereas HDL cholesterol is more strongly associated with the incidence of a first coronary heart disease event. Weverling-Rijnsburger et al. (1997) concluded that in people older than 85 years, high total cholesterol concentrations are associated with longevity owing to lower mortality from cancer and infection. Chyou and Eaker (2000) reported that an increased ratio of total cholesterol to high-density lipoprotein appears to be associated with an increase in risk for all-cause mortality in men aged 65 and over, while an elevated level of high-density lipoprotein, considered alone, seems to be protective against mortality from all causes in men aged 65–74 years, but this effect diminishes over the age of 75. Karlamangla et al. (2004) reported that increases in cholesterol over time have beneficial associations in some older adults. The authors concluded that the role of cholesterol changes in the health of older individuals needs further exploration. Upmeier et al. (2009) concluded that high levels of serum total cholesterol and particularly low levels of HDL-C seem to be risk factors for cardiovascular mortality even in the elderly population. Newson et al. (2011) found that higher total cholesterol was associated with a lower risk of non-cardiovascular mortality in older adults. This association varied across the late-life span and was stronger in older age groups. The authors concluded that further research is required to examine the mechanisms underlying this association.

Several recent studies in Japan reported that all-cause mortality among individuals with the highest total cholesterol levels was lower than in the other individuals (Ogushi and Kurita, 2008; Noda et al., 2010; Nago et al., 2011; Hamazaki et al., 2012a). Hamazaki et al. (2012b) reported that almost all epidemiological studies in Japan showed that all-cause mortality was lower in subjects with high levels of total cholesterol. Our results in Figure 3 for age trajectories of SCH levels among long-lived and short lived individuals support Japanese finding. The biological mechanisms responsible for such connection require separate study.

### Genes influencing lifespan may be a mix of variants favoring extreme longevity and health

A comparative look at Figures 3, 5, and 6 indicates that the 27 SNP alleles associated with lifespan influence the age trajectories of physiological indices in this study in complex ways involving both health and aging related processes. This is because all types of the effects seen in Figures 3 and 5 appear in the Figure 6, including the parallel shift of the age trajectory of an index to the right (for BMI); the same age at the peak value but different levels of an index at the peak (for DBP and SCH); and different rates of decline in the index value at older ages (for VR). This means that some of the 27 pro-survival alleles may modulate predisposition to particular diseases, some may regulate the rate and onset of physiological aging changes, and some other may have pleiotropic influence on respective traits. These results are in agreement with our earlier analysis of the functional effects of genes linked to the 27 SNPs, where we found that such genes are involved in both physiological aging and common diseases (Yashin et al., 2012c).

Note that we did not match the (<14)- and (≥14)-groups of the study subjects for other confounding factors. This is because we would like to show how age trajectories of physiological indices differ for individuals carrying different numbers of “pro-survival” genetic variants. The age patterns of physiological change for human individuals with different genetic background have never been studied before. These results do not pretend to be interpreted as causal relationships. They, however, help researchers get useful insights and ideas on how such relationship could be evaluated in future studies. The effects of genetic and non-genetic factors on dynamics of each physiological variable deserve separate analyses.

### Non-monotonic changes in physiological indices

Our analyses showed that the population average trajectories of BMI, DBP, SCH, and VR, as well as their average biological age trajectories (at least for the LL individuals), are non-monotonic. This property of aging-related changes may reflect the decline in functioning of various tissues and organs involved in the regulation of these variables. This means that the difference in the ages at which a given variable reached its maximum value in different groups of individuals might provide us with useful information about the rates of aging in these groups. The links of this parameter with lifespan and healthy lifespan were established in Yashin et al. (2010a). Several other characteristics of dynamic behavior of age trajectories of biological indices were also associated with lifespan. These include the average values, as well as the slopes and intercepts of physiological indices calculated between ages 40 and 60 years, the value of the maximum, the rate of decline after reaching the maximum value and the individual variability of an index during the life course (Yashin et al., 2006, 2010a).

The non-monotonic age patterns of physiological variables present additional challenges for those who seek biomarkers of aging capable of measuring “biological” age. The indices investigated above are not good for that. This is because of the non-linear changes of their values with age, e.g., they can be about the same at ages 45–50 and 85–90 years. It is important to note that the forces driving the average age trajectories of physiological variables cannot be detected from the data using standard statistical methods. These mechanisms, however, can be described and their characteristics can be estimated from the data using advanced methods of statistical modeling, see Yashin et al. (2012a) and references therein. Two possible mechanisms contributing to corresponding shapes have been proposed (Yashin et al., 2010b). The first one deals with biological homeostatic regulation of the values of physiological variables in response to aging related changes, and to persistent external challenges. This mechanism is represented by the first term in Equation (3). The second mechanism deals with mortality selection in heterogeneous populations in which individuals with high deviations of physiological variables from the norm have substantially higher mortality risks. The deceased individuals, as well as those who leave the study for other reasons at some age, drop out of the averaging procedure after this age. This part of the mechanism is represented by the second term in the Equation (3).

To evaluate the shape of the biological age trajectories without compositional changes and to compare whether the age trajectories of individuals having long lifespan differ from those who died prematurely, we divided members of the Original FHS cohort into the two sub-cohorts of the SL and LL individuals. The LL individuals included all those whose lifespan exceed 97 years. The results of our analyses indicated that biological age trajectories of the four physiological variables studied in this paper were non-monotonic. Typically the SL persons had initially higher average values of these indices. The average age trajectories for the SL persons reached their maximum values, and started to decline earlier than those of the LL persons. The average age trajectories of the LL persons describe their average biological changes until about age 97, and these changes were non-monotonic. The fact that these trajectories differed substantially for the SL and LL groups of individuals indicates that they contain useful information about remaining lifespan distributions which could be used for predicting this trait.

### Intersection of the age trajectories for healthy and unhealthy persons

The fact that average age trajectories of DBP, SCH, and VR for healthy and unhealthy individuals intersect may indicate fundamental aging related changes occurring on the way from the old to the oldest old ages. Such a transition is likely to rearrange factors of susceptibility to diseases and change the role of risk factors from deleterious to neutral or even favorable. These intersections, however, do not explain what kind of forces might be responsible for such behaviors of these curves. One possibility is that the genetic factors could contribute to observed patterns.

The genetic analyses of the age trajectories of the four indices indicate that the genetic influence of pro-survival alleles may differ from one index to the next. For example, the effect of the (<14)- and (≥14)-groups on BMI differs from those on DBP and VR, and the effects of these genetic groups on SCH are similar to these two but the effects on SCH become more pronounced only after age 50. This indicates that studying the roles of genes in aging related changes requires more information about biological mechanisms involved in regulation of these characteristics. Analyses of genetic influence on age patterns of average physiological indices for healthy and unhealthy individuals resulted in similar conclusions. They also showed that the selected pro-survival alleles were likely to contribute to mortality from CVD and cancer.

### The use of stochastic process model in analyses of longitudinal data

The statistical analyses of longitudinal data using stochastic process model allowed us to evaluate hidden dimensions of aging related changes which are important for better understanding regulatory mechanisms driving observed aging related changes in physiological variables. The hidden components of aging related changes incorporated into our model include adaptive capacity, resistance to stresses, physiological norm, and the effect of allostatic adaptation [see notations for variables used in Equations (1) and (2)]. All these variables play important role in the aging process but were not directly measured in longitudinal data. The adaptive capacity characterizes the ability of organisms to keep the values of physiological variable around set-point of homeostatic regulation. The higher absolute values of this variable correspond to better organism's capacity to adapt and provide it with better fitness. Physiological norm describes optimal value of physiological variable which minimizes mortality risk. The resistance to stresses characterizes sensitivity of mortality risk to the deviation of physiological variable from the norm. The effect of allostatic adaptation characterizes set-point in mechanism of homeostatic regulation of physiological index. This component of physiological change integrates influence of persistent external disturbances. Note that since external disturbances are not measured in most of longitudinal studies, the estimates of the difference between this variable and physiological norm (allostatic load) may serve as an important indicator of healthy or unhealthy environment individuals under study are exposed to. Our analyses show that these characteristics can be estimated from the data using stochastic process model of human mortality and aging. The use of such model allows for testing statistical hypotheses not only about genetic influence on age trajectories of physiological indices but also about the roles of genes in mechanisms involved in regulation of these trajectories.

## Conclusions

The age trajectories of physiological indices corresponding to individuals with exceptional longevity differ from those of people living without three major human diseases—cancer, CVD, and diabetes. These results may indicate that factors responsible for the long life and good health are not necessary the same. The trajectories for long-living individuals look as if their age related changes were postponed compared to persons died prematurely.

The analyses confirmed that genetic influences on lifespan are realized through dynamic mechanisms regulating changes in physiological variables during the life course as well as through variables describing individual health status. The genetic dose index constructed from genetic variants selected for their individual associations with lifespan showed association with mortality rates by cause. This indicates that these genetic factors influence both health and lifespan.

The average aging related changes in the four selected physiological variables are likely to be driven by hidden components of aging changes and by genetic factors.

The ability of advanced methods of statistical modeling to estimate hidden components of aging changes in humans indicates that the approach can be further extended to perform more comprehensive analyses of available data by incorporating relevant biological knowledge about aging into statistical models. The use of such models in statistical analyses of data will help researchers to untangle complex age-dependent dynamic relationships among biomarkers and elucidate roles of genes and non-genetic factors in aging, health, and lifespan.

### Conflict of interest statement

The authors declare that the research was conducted in the absence of any commercial or financial relationships that could be construed as a potential conflict of interest.
